# Short-Term Repeated-Sprint Training in Hot and Cool Conditions Similarly Benefits Performance in Team-Sport Athletes

**DOI:** 10.3389/fphys.2020.01023

**Published:** 2020-08-27

**Authors:** Julien D. Périard, David B. Pyne, David J. Bishop, Alice Wallett, Olivier Girard

**Affiliations:** ^1^Research Institute for Sport and Exercise, University of Canberra, Canberra, ACT, Australia; ^2^Institute of Health and Sport, Victoria University, Melbourne, VIC, Australia; ^3^School of Human Sciences (Exercise and Sport Science), The University of Western Australia, Perth, WA, Australia

**Keywords:** heat acclimation, thermoregulation, intermittent sports, running, repeated-sprint ability

## Abstract

This study compared the performance and physiological adaptations of short-term repeated-sprint training in HOT [40°C and 40% relative humidity (RH)] and COOL (20°C and 40% RH) conditions in team-sport athletes. Twenty-five trained males completed five training sessions of 60 min over 7 days in HOT (*n* = 13) or COOL (*n* = 12) conditions, consisting of a submaximal warm-up and four sets of maximal sprints. Before and after the intervention, intermittent shuttle running performance was assessed in cool and repeated-sprint ability in hot conditions; the latter preceded and followed by neuromuscular function testing. During the repeated-sprint training sessions, skin (~8.4°C) and core (~0.17°C) temperatures were higher in HOT than COOL (*p* < 0.05) conditions. Shuttle running distance increased after both interventions (*p* < 0.001), with a non-significant (*p* = 0.131) but larger effect in HOT (315 m, *d* = 1.18) than COOL (207 m, *d* = 0.51) conditions. Mean (~7%, *p* < 0.001) and peak (~5%, *p* < 0.05) power during repeated-sprinting increased following both interventions, whereas peak twitch force before the repeated-sprint assessment was ~10% lower after the interventions (*p* = 0.001). Heart rate during the repeated-sprint warm-up was reduced (~6 beats.min^−1^) following both interventions (*p* < 0.01). Rectal temperature was ~0.14°C lower throughout the repeated-sprint assessment after the interventions (*p* < 0.001), with larger effects in HOT than COOL during the warm-up (*p* = 0.082; *d* = −0.53 vs. *d* = −0.15) and repeated-sprints (*p* = 0.081; *d* = −0.54 vs. *d* = −0.02). Skin temperature (*p* = 0.004, *d* = −1.11) and thermal sensation (*p* = 0.015, *d* = −0.93) were lower during the repeated-sprints after training in HOT than COOL. Sweat rate increased (0.2 L.h^−1^) only after training in HOT (*p* = 0.027; *d* = 0.72). The intensive nature of brief repeated-sprint training induces similar improvements in repeated-sprint cycling ability in hot conditions and intermittent running performance in cool conditions, along with analogous physiological adaptations, irrespective of the environmental conditions in which training is undertaken.

## Introduction

Exercise-heat acclimation is used by endurance athletes to optimize performance when competing in the heat. The improvements in performance are attributed to enhanced sweating and skin blood flow responses, plasma volume expansion, increased cardiovascular stability, better fluid balance, and acquired thermal tolerance (Rowell, 1974; Sawka et al., 2011; Périard et al., 2015). The four main approaches conferring these adaptations include constant work rate exercise (e.g., 60% peak oxygen consumption: $\dot{V}$O_2peak_), self-paced exercise (i.e., variable work rate exercise), controlled hyperthermia (i.e., attain and maintain a target core temperature), and controlled heart rate (i.e., attain and maintain a target heart rate) heat acclimation (Daanen et al., 2018). The exercise associated with these approaches is typically endurance-based (i.e., low to moderate-intensity exercise; Nadel et al., 1974; Pandolf et al., 1977; Nielsen, 1998), with daily exposures of 60–90 min undertaken for 10–14 days (Nielsen et al., 1993; Lorenzo et al., 2010; Racinais et al., 2015).

The training required to optimize performance and heat tolerance in team-sport athletes, however, may differ to that commonly prescribed for endurance athletes. For sports such as rugby sevens, soccer, and field hockey, high-level performance involves the ability to repeat maximal or near-maximal sprint efforts (Girard et al., 2015). For example, a defining characteristic of international soccer players is their ability to perform ~60% more sprinting than professional players of a lower standard (Mohr et al., 2003). When playing under heat stress, however, repeated-sprinting and jumping ability are compromised to a greater extent than when playing in temperate conditions (Mohr et al., 2010). This compromise occurs despite evidence that players adjust their physical activity patterns (e.g., decrease total and high-intensity running distance) in the heat to maintain the capacity to perform periodic sprint efforts when required at key moments in a match (i.e., pacing; Duffield et al., 2009; Mohr et al., 2012; Nassis et al., 2015). Repeated-sprint heat acclimation may thus be an approach that minimizes the impact of hot environmental conditions on repeated-sprint ability and low-to-moderate intensity exercise, whereby the pace and physical activity patterns adopted by team-sport athletes can be maintained throughout a match.

At the elite level, the training time and travel constraints of team-sport athletes are such that prolonged (>7 days) interventions may be difficult to implement during the regular season (Chalmers et al., 2014). Short-term heat acclimation regimens involving repeated sprints may therefore be more appropriate, particularly since repeated-sprinting improves physical performance (i.e., repeated-sprint ability; Bishop et al., 2011), and short-term heat acclimation can initiate thermoregulatory adaptations and plasma volume expansion and improve perceptions of exertion and thermal comfort (Garrett et al., 2009, 2011; Chalmers et al., 2014). The distinction between repeated-sprint and intermittent or high-intensity interval training is important and lies with the intensity and length of the efforts, as well as the duration of recovery between efforts. Repeated sprinting is characterized by brief “all-out” efforts (≤10 s) and incomplete recovery (≤60 s), whereas intermittent sprinting involves longer recovery periods (60–300 s; Girard et al., 2011). High-intensity interval training involves short-to-long efforts (45–240 s) of high but not maximal intensity exercise, interspersed with recovery periods or varying length (e.g., work/rest ratio of 1:1 or more; Buchheit and Laursen, 2013).

Although repeated-sprint ability is regarded as a key performance determinant for team-sport success (Girard et al., 2011; Billaut et al., 2012; Sweeting et al., 2017), only one study appears to have used maximal (i.e., “all-out”) repeated sprinting to promote heat adaptation over a short time frame (<7 days). Petersen et al. (2010) had participants complete maximal cycling sprints on 4 consecutive days in 30°C and 61% relative humidity (RH) for a total heat exposure time of 150 min. This heat training intervention decreased heart rate and thermal discomfort and improved thermal sensation at the end of a 30-min submaximal running test in the heat, relative to participants who trained in 20°C and 63% RH. However, short-term heat training did not influence repeated-sprint performance in temperate conditions (24°C and 48% RH). A more protracted maximal repeated-sprint protocol performed in 35°C and 60% RH conditions on either 8 consecutive days or every second day, yielded a similar adaptive response in thermoregulatory capacity and improvements in repeated-sprint performance, regardless of the approach (i.e., continuous or intermittent; Duvnjak-Zaknich et al., 2019). The improvements in performance were maintained for 2 weeks following completion of both the continuous and intermittent protocols, which provided a total exercise-heat exposure time of 324 min with a daily change in core temperature of 1.3–1.8°C. In contrast, six sessions of Wingate interval training over 2 weeks in 40°C did not improve $\dot{V}$O_2peak_ in cool conditions nor induce heat adaptation when assessed in 25°C conditions (Wingo et al., 2018). The authors concluded that the overall exposure time (165 min) and minimal increase in daily core temperature (0.6–0.9°C) were insufficient to promote an adaptive response, as profuse sweating and elevated skin and core temperatures are required to induce adaptations (Sawka et al., 2011; Périard et al., 2015). Altogether, it appears that heat adaptations and performance improvements with short-to-medium-term maximal sprint heat acclimation protocols relate to both the magnitude and total duration of exercise-heat exposure, as well as the environmental conditions in which performance tests (e.g., aerobic capacity and repeated-sprint ability) are completed. The performance benefits may also be influenced by the fatiguing nature of the exercise and a lack of adequate recovery between training sessions and subsequent testing (Reeve et al., 2019).

The purpose of this study was to determine whether five sessions of repeated-sprint training over 7 days (2 days of recovery) in hot environmental conditions (40°C and 40% RH) induced adaptations commensurate with heat acclimation and greater improvements in repeated-sprint ability and aerobic performance than training in cool conditions (20°C and 40% RH). To elucidate the potential pathway *via* which performance may be enhanced or influenced by cumulative muscle fatigue, a neuromuscular function assessment was also conducted. It was hypothesized that repeated-sprint training with 300 min of total heat exposure would initiate a heat acclimation response and enhance both repeated-sprint ability in the heat and aerobic performance in cool conditions.

## Materials and Methods

### Participants

Twenty-five trained, non-heat acclimatized (June to November in Canberra, average high temperature of 15.5°C), male team-sport athletes (regional level soccer, rugby, and Australian rules football) training ~7 h per week participated in the study. Thirteen participants completed the training regimen in HOT conditions – age, body mass, height, and $\dot{V}$O_2peak_: 26 ± 5 year, 81.9 ± 9.7 kg, 1.8 ± 0.1 m, and 50.7 ± 3.9 ml.kg^−1^.min^−1^ (mean ± SD), respectively. Twelve participants completed the training regimen in COOL conditions – 23 ± 4 year, 81.7 ± 10.5 kg, 1.8 ± 0.1 m, and 51.8 ± 5.9 ml.kg^−1^.min^−1^. Participants were fully informed of the experimental procedures and potential risks prior to giving written informed consent. All participants completed an Adult Pre-exercise Screening Tool (Exercise & Sport Science Australia, Ascot, Australia, 2011) before admission to the study. The protocol was approved by the University of Canberra Human Research Ethics Committee (17-115) and all procedures conformed to the standards of the Declaration of Helsinki.

### Experimental Design

**Figure 1:**
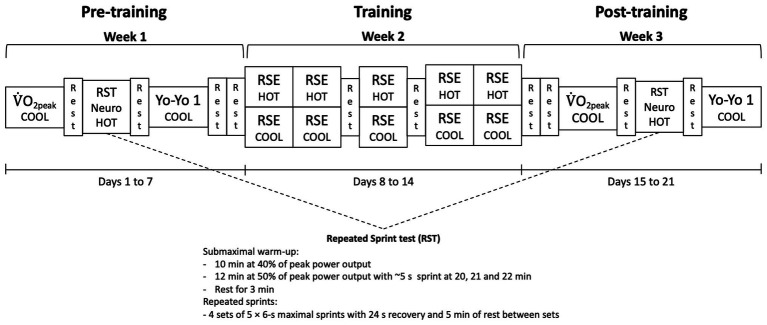
Experimental overview of the repeated-sprint exercise (RSE) training protocol undertaken by separate groups in either HOT (40°C and 40% RH) or COOL (20°C and 40% RH) conditions, as well as the pre‐ and post-training assessments performed in conditions as indicated. $\dot{V}$O_2peak_, peak oxygen consumption test; RST, repeated-sprint test; Neuro, neuromuscular function assessment; and Yo-Yo 1, intermittent recovery test level 1.

Participants visited the laboratory ~1 week prior to commencing the study for a familiarization session where the neuromuscular function assessment and repeated-sprint protocol were performed in cool conditions. Participants were then assigned *via* block randomization (Suresh, 2011) to either the HOT or COOL (i.e., control) training group in a counterbalanced order and undertook three pre-experimental tests, each separated by 48 h (Figure 1). The tests were repeated after the repeated-sprint training regimen and included a $\dot{V}$O_2peak_ test in cool conditions, a repeated-sprint test (RST) in the heat, which was immediately preceded and followed by a neuromuscular function assessment of the knee extensors in cool conditions, and a Yo-Yo Intermittent Recovery Test Level 1 (Yo-Yo IR1) conducted in cool conditions. After completing all pre-experimental tests, participants undertook a training regimen consisting of five sessions in 7 days in either the HOT or COOL condition. The regimen consisted of performing a repeated-sprint protocol on days 1, 2, 4, 6, and 7 (Figure 1). Temperature and RH were standardized for all procedures undertaken in the HOT (40°C and 40% RH) and COOL (20°C and 40% RH) conditions, except for the Yo-Yo IR1 test performed in 15–18°C. All testing and training sessions were conducted at the same time of day for each individual.

### Testing and Training Procedures

#### VO_2peak_ Test

The incremental exercise test to determine $\dot{V}$O_2peak_ consisted of cycling on an electromagnetically braked cycle ergometer (Velotron, RacerMate Inc., Seattle, WA, USA) at an initial power output of 100 W with a 25 W.min^−1^ increase until volitional fatigue. Expired air was analyzed by paramagnetic O_2_ and infrared CO_2_ analyzers (ParvoMedics Inc., Salt Lake City, UT, USA). Heart rate was monitored continuously with a Polar transmitter-receiver (T-31 Polar Electro, Lake Success, USA) and captured within the Velotron software.

#### Yo-Yo IR1 Test

The Yo-Yo IR1 was used as a team-sport-specific field test, consisting of repeating 20-m shuttle runs at increasing speeds, starting at 10 km.h^−1^. Each run is interspersed with an active recovery period of 10 s, consisting of 5 m of jogging/walking (Krustrup et al., 2003). Verbal encouragement was provided to participants when nearing the end of the test, which was conducted indoors on a synthetic running track.

#### Repeated-Sprint Test

On arrival to the laboratory (~60 min prior to testing), participants emptied their bladder and provided a urine sample for measurement of urine specific gravity (USG: PEN-Urine SG, Atago, Tokyo, Japan). If USG was >1.020, a 300 ml bolus of water was consumed within 10 min. Participants were then weighed before changing into sporting attire (shorts, socks, and shoes) and self-inserting a general-purpose thermistor probe (TM400, Covidien, Mansfield, MA, USA) 12 cm beyond the anal sphincter to measure rectal temperature (*T*_re_). Participants then rested in the seated position in cool conditions while instrumented with a heart rate monitor and skin temperature sensors (iButton, Maxim Integrated Products, San Jose, CA, USA) to calculate mean skin temperature (*T*_sk_; Ramanathan, 1964). Baseline measurements were taken after 5 min in the supine position prior to undertaking the neuromuscular function assessment. Participants then entered the climate chamber set to HOT conditions and mounted a cycle ergometer (Wattbike Pro, Nottingham, UK). After 2 min of seated rest, a 22 min warm-up was initiated, consisting of cycling at 40% of the peak power output achieved during the $\dot{V}$O_2peak_ test for 10 min, and then 50% of peak power output for the following 12 min. At 20, 21, and 22 min, a brief (~5 s) maximal sprint was performed. Participants then rested for 3 min.

The repeated-sprint protocol consisted of four sets of 5 × 6-s maximal (i.e., all-out and standing) cycling sprints separated by 24 s of passive recovery and 5 min of seated rest between sets. The sprints were conducted on the Wattbike ergometer at air and magnetic resistances of 10 and 3, respectively. A countdown to start and finish each sprint was provided, as well as strong verbal encouragement. Ratings of perceived exertion (RPE; Borg, 1982) and thermal sensation (American Society of Heating, Refrigeration and Air-Conditioning, 1966) were recorded at the end of each set. Constant airflow (~12.5 km.h^−1^) was provided by an electric fan aimed at the torso and head of the participants during the repeated-sprint protocol. Participants were permitted to drink water *ad libitum* during the repeated-sprint protocol. Change in body mass was calculated at the conclusion of the “Neuromuscular Function Assessment” to determine whole-body sweat production with corrections for fluid ingested, urine losses, and sweat trapped in clothing.

#### Neuromuscular Function Assessment

Following baseline measurements for the RST, a neuromuscular function assessment consisting of voluntary and electrically-evoked contractions was performed with participants seated upright on a custom-built adjustable chair with the hips and knees flexed at 90°. Restraining straps placed across the chest and hips secured the participant in the chair and prevented extraneous movement, while a dynamometer (CAPTELS, Saint-Mathieu-de-Treviers, France) was attached 3–5 cm above the tip of the lateral malleoli. During all contractions, the force signal was amplified, sent through an A/D board, and sampled at 2,000 Hz by commercially available hardware and software (Biopac Student Lab 4.1, BIOPAC Systems Inc., Santa Barbara, California, USA). A high-voltage stimulator (Digitimer DS7AH, Digitimer, Welwyn Garden City, UK) was used to deliver a square-wave stimulus of 0.2 ms duration with a maximal voltage of 400 V. The femoral nerve was stimulated by placing a cathode (5 mm diameter) in the inguinal crease and an anode (5 × 9 cm TENS Pads, TENS Machines, West End, Queensland, Australia) in the gluteal fold. During the familiarization session, an isometric recruitment curve using motor nerve stimulation was drawn on the relaxed quadriceps to individualize the stimulus intensity. The current was increased in 20 mA increments until a plateau occurred in maximal twitch amplitude. Supramaximal stimulation for the experimental trials was ensured by increasing the final intensity by 50% (HOT: 205 ± 73 and COOL: 227 ± 27 mA; mean ± SD). The neuromuscular assessment consisted of a brief (5 s) maximal voluntary isometric contraction (MVC) of the knee extensors, on which a paired stimulus (doublet at 100 Hz) was superimposed. This contraction was followed by another paired stimulus (i.e., potentiated doublet) and a single pulse (i.e., twitch) on the relaxed muscle, each interspersed by 5 s. During all MVCs, the participants were instructed to reach maximal force as quickly as possible and maintain this level for the duration of the contraction. Participants were strongly encouraged with verbal reinforcement and a visual display of force production. Approximately 30 s after completing the final sprint of the RST, participants made their way to the neuromuscular function chair where they repeated the neuromuscular function assessment.

#### Repeated-Sprint Training

Each session of the repeated-sprint training regimen was conducted as outlined above, with one group training in HOT and the other in COOL conditions. The training sessions did not include a neuromuscular function assessment. However, a 10-min cool-down at 40% of $\dot{V}$O_2peak_ power output was added to extend total trial duration of each session to 60 min (including the 25 min warm-up). Throughout testing and training, the participants were asked to maintain a normal diet, but avoid the consumption of alcohol for 24 h and caffeine for 8 h prior to testing or training. Participants were also instructed to avoid strenuous training outside of the study protocol, maintain their usual aerobic/endurance training sessions, and complete a daily training diary, which was verified upon arrival for training.

### Data Analysis

Repeated-sprint performance was analyzed by calculating mean and peak power output across each set. The percent decrement score {% = [1 − (sprint 1 + 2 + 3 + 4 + 5)/best sprint × number of sprints] × 100} was calculated from the mean and peak power outputs recorded across each set (Girard et al., 2011). The neuromuscular function analysis was performed using the Biopac software. Voluntary activation was calculated with the interpolated twitch technique {VA (%) = [1 − (superimposed doublet/resting potentiated doublet)] × 100}. Force production in the calculation of voluntary activation was recorded as the average force of the 250 ms period prior to motor nerve stimulation. Peak twitch force was assessed from the resting twitches evoked after the MVCs. The highest of the three brief MVCs was selected to assess voluntary force production and activation, whereas the mean of the three twitches was used to analyze peak twitch force.

### Statistical Analysis

An *a priori* power analysis for sample size estimation was conducted for changes in resting core temperature following short-term heat acclimation based on two studies with a partial eta-squared of 0.27 (Moss et al., 2019) and 0.10 (Reeve et al., 2019). Power analyses were also performed for changes in repeated-sprint performance with a partial eta-squared of 0.32 (Duvnjak-Zaknich et al., 2019) and Yo-Yo IR2 performance with a partial eta-squared of 0.92 (Racinais et al., 2014). With an alpha of 0.05 and power of 0.80, the estimated sample size needed for repeated measures of within and between group comparisons was 8 and 20 for resting core temperature, and 8 and 4 for repeated-sprinting and Yo-Yo IR2 performance (G*Power 3.1.9.6). Given the nature of the project and the potential for abandonment, 13 participants (one subsequent abandonment) per group were recruited to ensure that the study was well powered. A mixed linear modeling procedure was used to estimate means (fixed effects) and within‐ and between-subject variations (random effects, modeled as variances). The fixed effects were the underlying thermal condition in which training occurred (HOT or COOL), the time at which testing was conducted relative to the intervention (pre‐ or post-intervention), and the time within a testing session. The random effects were between-subject variances in performance, perceptual, and thermal adaptations, whereas within-subject variances represented typical variation in adaptation over the five training sessions. Where significant effects were established, pairwise comparisons were identified using the Bonferroni *post hoc* analysis adjusted for multiple comparisons. The rest, warm-up, and sprint segments of the RST were analyzed separately as they corresponded to periods of rest, submaximal constant work rate exercise, and maximal exercise, respectively. Paired sample *t*-tests were conducted to compare the within-group treatment (i.e., pre-to-post training intervention) effects on markers of hydration status (i.e., baseline body mass and USG). Model parameters and effects are reported as mean with 95% confidence interval (CI: lower and upper bound) unless otherwise indicated. Cohen’s *d* effect sizes were interpreted as small (0.2), medium (0.5), and large (0.8; Cohen, 1992). All statistical analyses were performed using SPSS Software (IMB SPSS Statistic Version 25).

## Results

### Training Data

*T*_re_ during the repeated-sprint and cool-down segments (35 min) of the training sessions averaged 38.03°C (37.95–38.20) in COOL and 38.20°C (38.13–38.67) in HOT (*p* = 0.002), whereas *T*_sk_ was 27.51°C (26.82–28.05) in COOL and 35.88°C (35.57–36.55) in HOT (*p* < 0.001). The greater overall thermal strain was accompanied by a higher heart rate during these segments in HOT [169 beats.min^−1^ (166–172)] compared with COOL [159 beats.min^−1^ (157–161); *p* < 0.001], and greater thermal sensation in HOT [6.3 (6.2–6.4)] than COOL [4.3 (4.1–4.6); *p* < 0.001].

### VO_2peak_ Test

$\dot{V}$O_2peak_ values were similar between groups [mean: 51.9 ml.kg^−1^.min^−1^ (49.3–54.6); *p* = 0.563], with no significant improvement following the intervention [1.2 ml.kg^−1^.min^−1^ (−0.3–2.6); *p* = 0.115; *d* = 0.24]. Maximum heart rate during the $\dot{V}$O_2peak_ test was similar between groups [mean: 188 beats.min^−1^ (184–191); *p* = 0.815] and decreased by 3 beats.min^−1^ (1–5) after the training intervention (*p* = 0.007; *d* = −0.31).

### Yo-Yo IR1 Test

**Figure 2:**
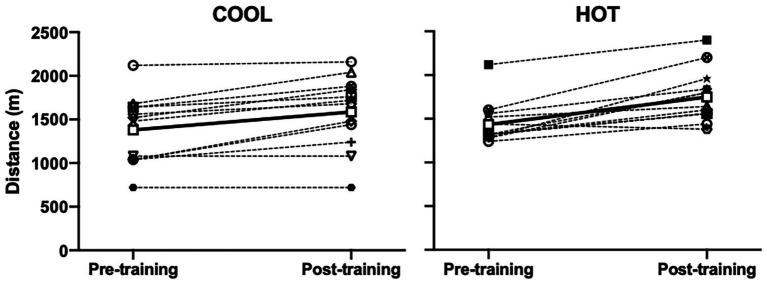
Individual and mean Yo-Yo intermittent recovery test (level 1) distance covered prior to (pre-training) and following (post-training) a repeated-sprint training intervention undertaken in COOL (20°C and 40% RH) or HOT (40°C and 40% RH) conditions. Open squares and solid lines are means within each group. Symbols omitted for clarity. Post‐ vs. pre-intervention main effect (*p* < 0.001, *d* = 0.78).

Distance covered in the Yo-Yo IR1 test was similar between groups prior to [HOT: 1,434 m (1,248–1,619); COOL: 1,380 m (1,187–1,573)] and following [HOT: 1,749 m (1,564–1,934); COOL: 1,587 m (1,394–1,780)] the intervention (Figure 2; *p* = 0.399). After the training intervention, a 261 m (189–333) increase in Yo-Yo IR1 distance occurred (*p* < 0.001; *d* = 0.78). The improvement in distance was not significantly different between the HOT and COOL groups [109 m (−35–252); *p* = 0.131], although the HOT group increased by 315 m (216–414; *d* = 1.18) and the COOL group by 207 m (103–309; *d* = 0.51).

### Repeated-Sprint Test – Physiological and Perceptual Responses

**Figure 3:**
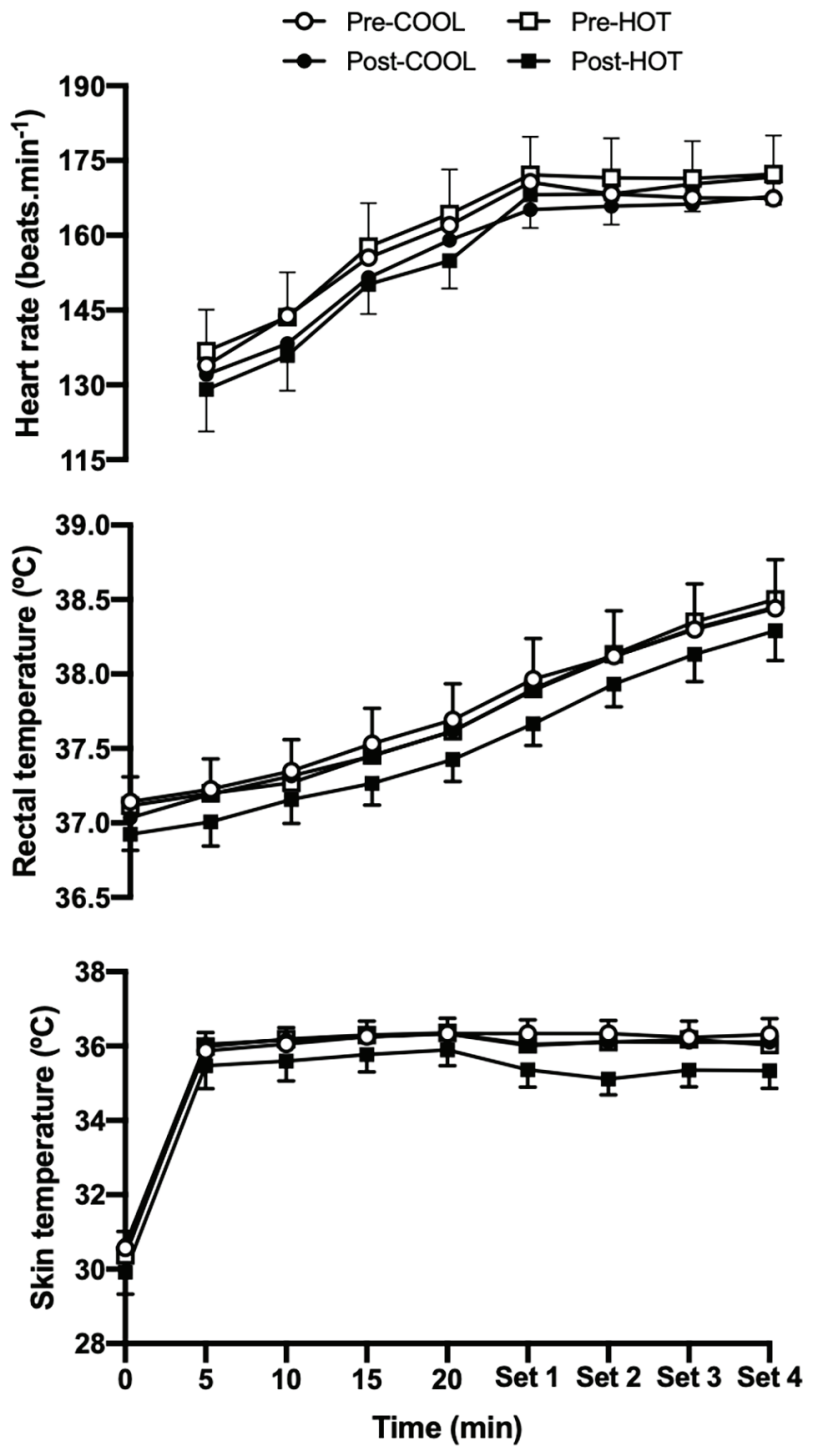
Heart rate, rectal temperature, and mean skin temperature during a RST including a 22 min submaximal warm-up and four sets of repeated sprints performed in 40°C and 40% RH prior to and following a repeated-sprint training intervention undertaken in COOL (20°C and 40% RH) or HOT (40°C and 40% RH) conditions. Data are mean with 95% CI. Symbols omitted for clarity. Heart rate: post‐ vs. pre-intervention main effect (warm-up: *p* < 0.001, *d* = −0.35; sprints: *p* < 0.001, *d* = −0.24). Rectal temperature: post‐ vs. pre-intervention main effect (warm-up: *p* < 0.001, *d* = −0.33; sprints: *p* < 0.001, *d* = −0.26). Skin temperature: post‐ vs. pre-intervention within condition effect for HOT (warm-up: *p* < 0.001, *d* = −0.59; sprints: *p* < 0.001, *d* = −1.03).

Resting heart rate was similar between the HOT [60 beats.min^−1^ (55–66)] and COOL [65 beats.min^−1^ (60–70)] groups prior to the intervention (*p* = 0.229), decreasing by 7 beats.min^−1^ (4–10) after the intervention (*p* < 0.001; *d* = −0.73). During the warm-up segment of the RST, an increase in heart rate occurred as a function of time (Figure 3; *p* < 0.001; *d* = 1.94), with mean heart rate 8 beats.min^−1^ (6–11) lower in the HOT group (*p* < 0.001; *d* = −0.49) and 4 beats.min^−1^ (1–6) lower in the COOL group (*p* = 0.006; *d* = −0.22) following the intervention. The magnitude of decrease in heart rate post-intervention was not significantly different between groups (*p* = 0.591; *d* = −0.15). Throughout the repeated-sprint segment of the RST, heart rate was 2 beats.min^−1^ (1–4) lower after completing the training intervention (*p* < 0.001; *d* = −0.24).

Resting *T*_re_ was similar prior to [37.1°C (37.0–37.2)] and following [37.0°C (36.9–37.1)] the training intervention in both groups (Figure 3; *p* = 0.081; *d* = −0.46). An increase in *T*_re_ was observed throughout the warm-up segment of the RST (*p* < 0.001; *d* = 1.36), with mean *T*_re_ lower by 0.11°C (0.05–0.16) after the training intervention (*p* < 0.001; *d* = −0.33). Although the lower *T*_re_ observed following the training intervention was not significantly different between groups, a larger standardized effect was observed in the HOT (~0.17°C) compared with COOL (~0.08°C) group at rest (*p* = 0.670; *d* = −0.64 vs. *d* = −0.31), during the warm-up (*p* = 0.082; *d* = −0.53 vs. *d* = −0.15) and repeated-sprint (*p* = 0.081; *d* = −0.54 vs. *d* = −0.02) segments.

Resting *T*_sk_ was similar prior to [30.5°C (30.0–30.9)] and following [30.3°C (29.9–30.7)] the training intervention in both groups (Figure 3; *p* = 0.355). During the warm-up segment of the RST, an increase in *T*_sk_ was observed (*p* < 0.01; *d* = 0.47), which following the training intervention was lower by 0.6°C (0.4–0.7) in the HOT group (*p* < 0.001; *d* = −0.59) and higher by 0.2°C (0.0–0.4) in the COOL group (*p* = 0.015; *d* = 0.09). There was a decrease in *T*_sk_ during the repeated-sprint segment of the RST in both the HOT (*p* < 0.001; *d* = −1.03) and COOL (*p* = 0.010; *d* = −0.34) groups following the training intervention, with a 0.8°C (0.3–1.3) greater reduction in the HOT group (*p* = 0.004; *d* = −1.11).

**Figure 4:**
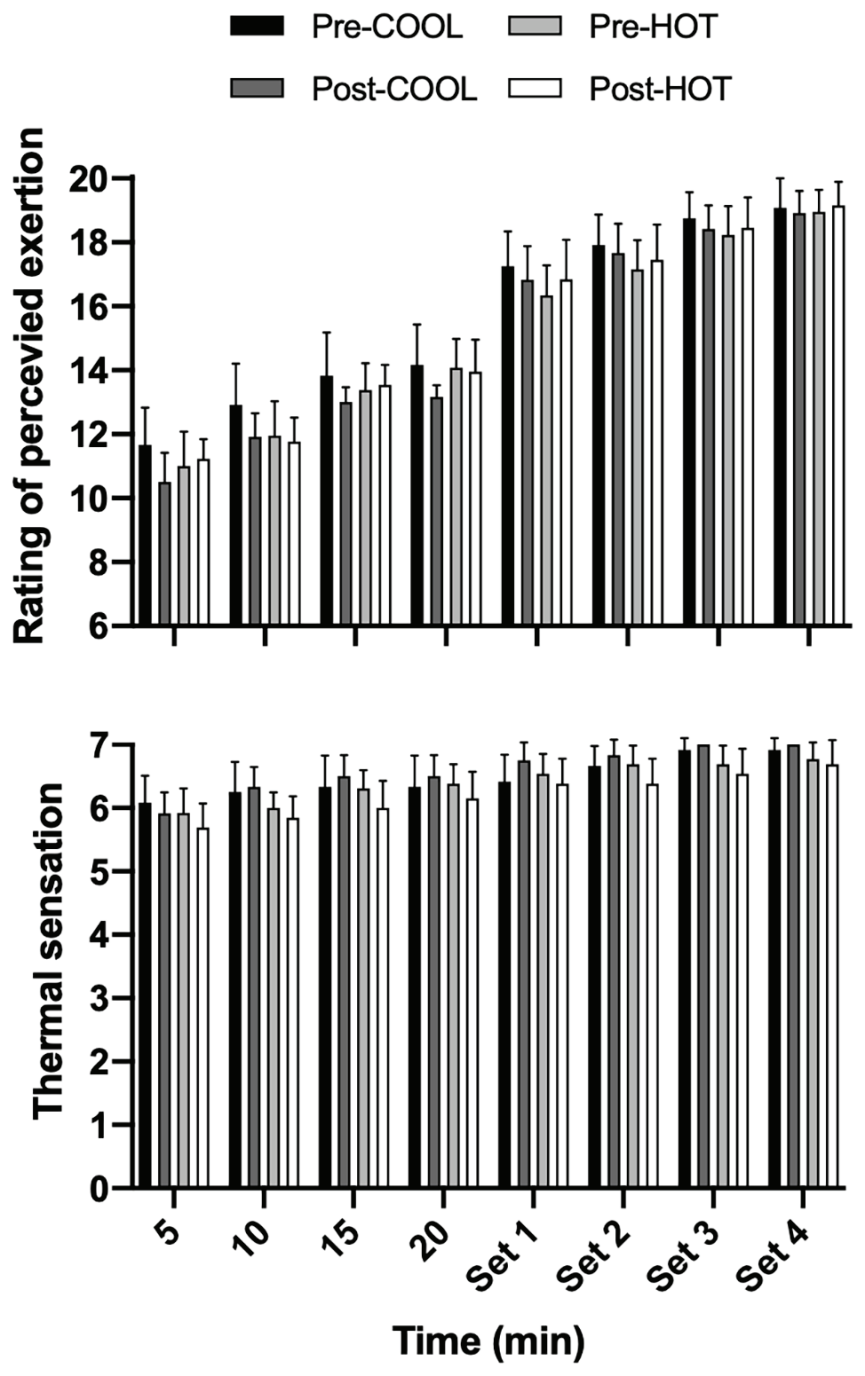
Rating of perceived exertion and thermal sensation during a RST including a 22 min submaximal warm-up and four sets of repeated sprints performed in 40°C and 40% RH prior to and following a repeated-sprint training intervention undertaken in COOL (20°C and 40% RH) or HOT (40°C and 40% RH) conditions. Data are mean with 95% CI. Symbols omitted for clarity. Thermal sensation: post-intervention HOT and COOL between condition effects (warm-up: *p* < 0.016, *d* = −0.81; sprints: *p* < 0.015, *d* = −0.93).

RPE increased throughout the warm-up segment of the RST in both groups (*p* < 0.001; *d* = 1.87), with a decrease of 1.0 unit (0.6–1.4) noted following the COOL intervention (*p* < 0.001; *d* = −0.55), but not the HOT intervention (*p* = 0.928; *d* = 0.01; Figure 4). There was also an increase in RPE during the repeated-sprint segment of the RST (*p* < 0.05; *d* = 1.50), with values remaining similar between groups following the intervention (*p* = 0.904). Thermal sensation at rest was unaffected by either intervention (*p* = 0.927). During the warm-up segment of the RST, an increase in thermal sensation was noted (*p* < 0.01; *d* = 0.78), which was lower by 0.5 units (0.1–0.8) following the training intervention in the HOT compared with COOL group (*p* = 0.016; *d* = −0.81). A similar response was observed during the repeated-sprint segment of the RST, where thermal sensation was lower by 0.4 units (0.1–0.8) in the HOT compared with COOL group after the training intervention (*p* = 0.015; *d* = −0.93).

### Repeated-Sprint Test – Performance Responses

**Figure 5:**
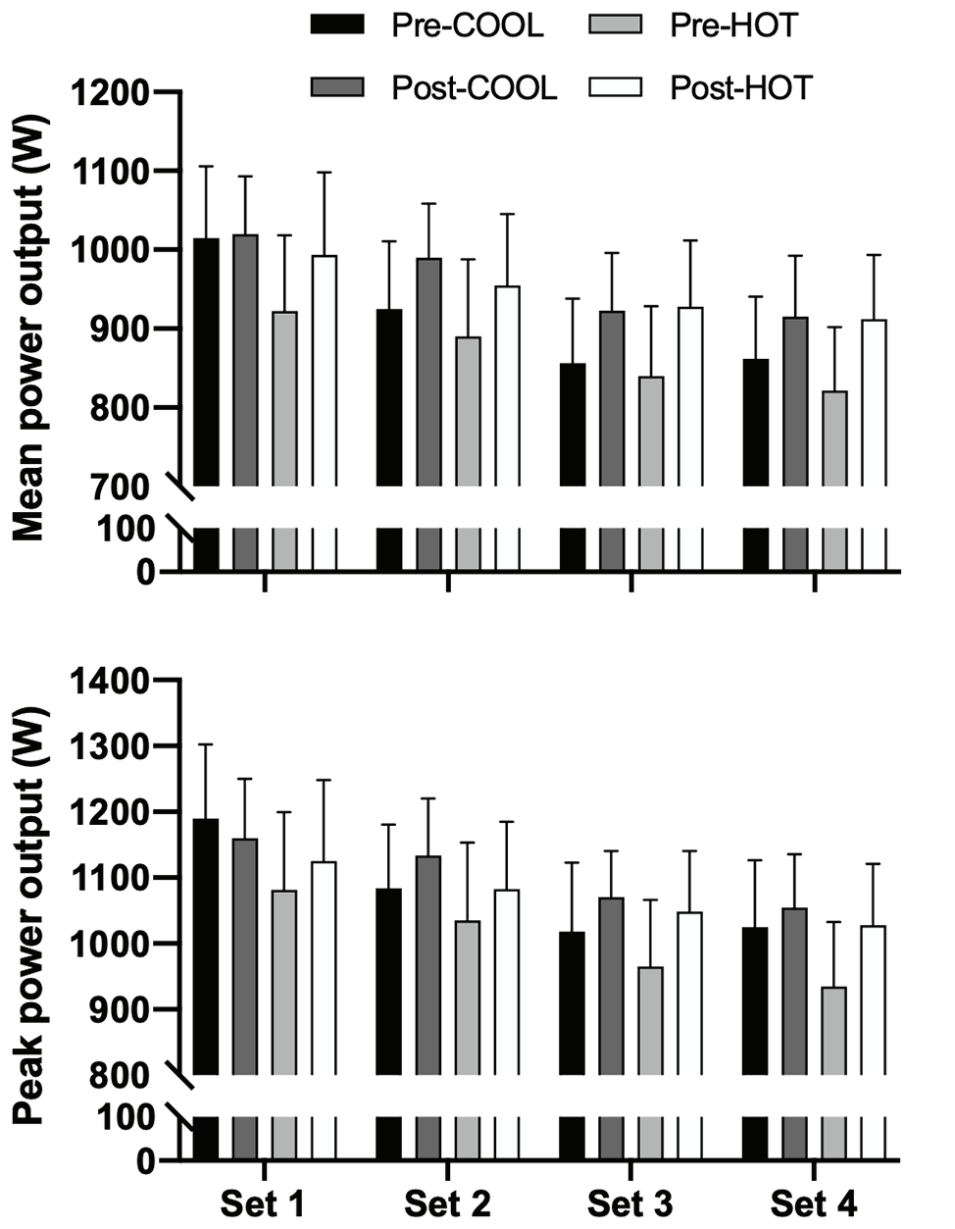
Mean and peak power output across sets during a four-set RST performed in 40°C and 40% RH prior to and following a repeated-sprint training protocol undertaken in COOL (20°C and 40% RH) or HOT (40°C and 40% RH) conditions. Data are mean with 95% CI. Symbols omitted for clarity. Mean power output: post‐ vs. pre-intervention main effect (*p* < 0.001, *d* = 0.45). Peak power output: post‐ vs. pre-intervention main effect (*p* < 0.001, *d* = 0.28).

Mean power across the RST was similar between groups (*p* = 0.547) but increased in both the COOL [48 W (27–68); *p* < 0.001; *d* = 0.36] and HOT [79 W (59–98); *p* < 0.001; *d* = 0.52] groups following the intervention (Figure 5). The percent decrement score for mean power was significantly different between groups (*p* = 0.030; *d* = 0.68). A significant decrease in the percent decrement score for mean power was observed following the intervention [2.2% (0.3–4.1); *p* = 0.025; *d* = −0.58]. Although not significantly different between the HOT and COOL groups [2.7% (−1.2–6.5); *p* = 0.167], the decrease in the percent decrement score was mostly due to the large [3.9% (1.0–6.8)] reduction in the COOL (*d* = −1.02) compared with HOT [1.2% (−4.2–1.7); *d* = −0.22] group. Peak power during the RST was similar between groups (*p* = 0.364) but increased following the intervention in the COOL [25 W (1.8–49); *p* = 0.035; *d* = 0.17] and HOT [67 W (44–89); *p* < 0.001; *d* = 0.38] groups. The percent decrement score for peak power was similar between groups (*p* = 0.445) and decreased after the intervention (*p* = 0.005; *d* = −0.67).

### Neuromuscular Function Assessment

A significant decrease in MVC force was evident following the RST (Table 1; *p* < 0.001; *d* = −1.32). Although force production capacity did not change after the training intervention (*p* = 0.888; *d* = −0.01), a slightly lower force production was noted Pre-RST after the training intervention [921 (862–982) to 896 N (835–958); *d* = −0.18]. Voluntary activation was unaffected by the RST (*p* = 0.289) or the intervention (*p* = 0.637). Peak twitch force decreased significantly Post-RST both prior to (*p* < 0.001; *d* = −2.72) and following (*p* < 0.001; *d* = −2.75) the interventions. Moreover, peak twitch force during the Pre-RST was lower after the training intervention (*p* = 0.001; *d* = −0.45).

**Table 1:** Maximal voluntary isometric contraction (MVC) force production, voluntary activation, and peak twitch force prior to (Pre) and following (Post) a repeated-sprint test (RST) in the heat.

		COOL		HOT		Main effects
		Pre-training	Post-training	Pre-training	Post-training	
MVC (N)	Pre-RST	924 (682–1,167)	911 (618–1,205)	920 (645–1,195)	878 (583–1,172)	COOL vs. HOT: *p* = 0.669, *d* = 0.15 Post-RST vs. Pre-RST: *p* < 0.001, *d* = −1.32 Post-training vs. Pre-training: *p* = 0.888, *d* = −0.01
	Post-RST	726 (515–938)	772 (506–1,038)	693 (424–962)	688 (402–973)	
Voluntary activation (%)	Pre-RST	92 (80–104)	93 (82–105)	95 (86–105)	95 (85–104)	COOL vs. HOT: *p* = 0.185, *d* = −0.34 Post-RST vs. Pre-RST: *p* = 0.289, *d* = −0.32 Post-training vs. Pre-training: *p* = 0.637, *d* = 0.10
	Post-RST	90 (74–106)	90 (69–111)	91 (70–112)	95 (82–107)	
Peak twitch force (N)	Pre-RST	182 (103–261)	167 (99–234)	169 (104–234)	151 (29–209)	COOL vs. HOT: *p* = 0.367, *d* = 0.39 Post-RST vs. Pre-RST: *p* < 0.001, *d* = −2.76 Post-training vs. Pre-training: *p* = 0.001, *d* = −0.19
	Post-RST	80 (28–132)	95 (66–124)	79 (32–126)	75 (32–118)	

The neuromuscular assessment was conducted before (Pre) and after (Post) a repeated-sprint training intervention undertaken in COOL [20°C and 40% relative humidity (RH)] or HOT (40°C and 40% RH) conditions. Data are mean (95% confidence interval).

### Hydration

Baseline body mass prior to and following the training intervention was similar within the COOL [82.1 kg (78.3–85.9); *p* = 0.266] and HOT [81.6 kg (77.8–85.4); *p* = 0.967] groups (values are means of pre‐ and post-intervention), as was USG [COOL: 1.014 (1.011–1.018); *p* = 0.435 and HOT: 1.018 (1.015–1.021); *p* = 0.167]. Sweat rate during the RST in the COOL group was unchanged following the training intervention [1.2 L.h^−1^ (1.1–1.4); *p* = 0.148; *d* = 0.41], whereas an increase occurred in the HOT group from pre [1.2 L.h^−1^ (0.8–1.5)] to post [1.4 L.h^−1^ (1.0–1.8)] intervention (*p* = 0.027; *d* = 0.72). Fluid intake during the RST remained unchanged by the training intervention in either the COOL [0.84 L (0.69–0.99); *p* = 0.447] or HOT [0.93 L (0.78–1.08); *p* = 0.413] group. Percent body mass losses during the RST were similar in the COOL [0.3% (0.1–0.5); *p* = 0.958] and HOT [0.1% (−0.1–0.3); *p* = 0.065; *d* = −0.53] groups prior to and after the intervention.

## Discussion

The aim of this study was to examine the physiological adaptations and performance changes associated with short-term repeated-sprint training in 40°C (HOT) compared with 20°C (COOL) conditions in team-sport athletes. Our data indicate the emergence of heat acclimation in those having trained in the heat, with a decrease in *T*_sk_ (~0.7°C) and thermal sensation (~0.5), along with an increase in whole-body sweat rate (0.2 L.h^−1^) during the post-intervention RST. Although not significantly different, slightly larger post-intervention improvements in *T*_re_ were noted in the HOT relative to the COOL group. From a performance perspective, $\dot{V}$O_2peak_ was unaffected by repeated-sprint training in either condition. In contrast, total running distance covered in the Yo-Yo IR1 test was increased in both groups (~18%), with a ~50% larger, albeit non-significant, post-intervention improvement observed in the HOT group. The repeated-sprint training intervention in both HOT and COOL conditions also improved repeated-sprint cycling ability in the heat (mean power: ~7%, peak power: ~5%). These improvements in performance occurred despite the presence of skeletal muscle fatigue in both groups, as demonstrated by a ~10% reduction in peak twitch force Pre-RST following the training intervention. These data indicate that five sessions of repeated-sprint training in HOT and COOL conditions over 7 days improved repeated-sprint ability in the heat and enhanced sport-specific endurance performance (i.e., 20-m shuttle run at increasing speeds) in cool conditions. From a physiological perspective, both training interventions induced an adaptive response, with partial heat adaptations emerging after training was conducted under heat stress. Taken together, these data indicate that the novel and intensive nature of a brief repeated-sprint training protocol induces analogous performance improvements and relatively similar physiological adaptations, irrespective of the environmental conditions in which it is undertaken.

### Adaptation to the Heat

Investigations into the adaptive response of short-term heat acclimation regimens have increased in recent years, particularly for their use in team sports (Sunderland et al., 2008; Chalmers et al., 2014; Reeve et al., 2019) as pre-season training camps, for tapering before competition, and as in-season performance-enhancing camps (Périard et al., 2015). Chalmers et al. (2014) suggested that five 60-min high-intensity sessions are required to improve aerobic performance in hot and potentially temperate environmental conditions in response to the development of cardiovascular, thermoregulatory, and metabolic adaptations. Data from previous short-to-medium term interval and repeated-sprint studies indicate that a sufficient thermal stimulus, both in terms of magnitude and duration, is required to promote the induction of heat acclimation (Petersen et al., 2010; Wingo et al., 2018; Duvnjak-Zaknich et al., 2019). For example, a 4-day program (total of 150 min) of repeated-sprint training in 30°C led to a lower heart rate and thermal perception at the end of a 30-min run in the heat; however, it did not elicit changes in core and skin temperature or sweat rate (Petersen et al., 2010). Conversely, an 8-day continuous or intermittent repeated-sprint protocol in 35°C led to an increase in sweat rate and the maintenance of a similar core temperature while cycling at a higher power output (Duvnjak-Zaknich et al., 2019). Several studies utilizing short-term high-intensity intermittent exercise (i.e., submaximal intervals with longer recovery) have also demonstrated evidence of partial heat acclimation (e.g., lower heart rate, decreased core and skin temperatures, improved thermal comfort, and increased sweat rate; Brade et al., 2013; Kelly et al., 2016; Schmit et al., 2018; Reeve et al., 2019). In the current study, total exposure time during the five repeated-sprint training sessions over 7 days was 300 min, with a daily increase in *T*_re_ and *T*_sk_ of ~1.6 and 6.5°C, respectively, in the HOT group, relative to an increase in *T*_re_ of ~1.2°C and decrease in *T*_sk_ of ~3.0°C in those training in COOL conditions. The increase in heart rate was also more pronounced in the HOT compared with COOL group during daily training, with ~77 vs. 70% and ~90 vs. 85% of maximum heart rate maintained during the warm-up and repeated-sprint segment of the RST, respectively. These responses indicate that a greater thermal stimulus for adaptation was provided to participants training in the heat.

In line with the provision of a greater thermal stimulus for adaption was an increase in sweat rate and reduction in *T*_sk_ during the entire RST after training in the HOT condition. These adaptations were coupled with non-significant, but larger standardized effects for a lower *T*_re_ (~0.1°C) across all phases of the RST (i.e., rest, warm-up, and repeated sprints; Figure 3) in the HOT compared with COOL condition. However, given the relatively small differences noted between conditions, it is debatable whether the adaptations noted in the heat had an appreciable physiological impact, despite improving thermal perception (Figure 4). The similar adaptive response observed between groups highlights the partial heat acclimation phenotype exhibited by athletes regularly training in cool conditions (Avellini et al., 1982; Armstrong and Pandolf, 1988), which could partly explain the lack of a greater difference in thermoregulatory improvements (Ravanelli et al., 2020), with this trained cohort requiring a greater thermal stimulus for adaptation. Although the current results indicate the emergence of thermoregulatory and perceptual adaptations in the HOT group, there appears to be potential for an extended repeated-sprint training regimen to more fully induce heat acclimation in trained individuals. Moreover, limiting convective airflow when training, particularly during the recovery segments between sprint sets, would induce greater thermal strain by reducing evaporative heat loss and potentially promote a greater adaptive response.

### Performance Improvement

The improvements in thermoregulatory capacity and cardiovascular stability afforded by heat acclimation are accompanied by attenuation of the deleterious impact of heat stress on aerobic performance (Périard et al., 2015; Tyler et al., 2016). In trained individuals, only five high-intensity sessions of 60 min may be required to improve aerobic-based performance in hot and cool conditions (Chalmers et al., 2014). However, there is disparity in the performance outcomes between studies utilizing this approach, which is inherently linked to the nature (i.e., repeated or intermittent sprint exercise) and brevity of the regimens (i.e., total duration of exposure), as well as the inter-individual variability in responsiveness. For example, five high-intensity interval training sessions over 9 days in 39°C decreased RPE and lactate concentration during submaximal exercise in the heat (Kelly et al., 2016). In well-trained females, a short-term high-intensity intermittent running regimen improved exercise capacity during the Loughborough Intermittent Shuttle Tests in 30°C heat by 33%, while maximal 15 m sprint time was unaffected (Sunderland et al., 2008). A tendency for improved work capacity in the latter part of an intermittent-sprint protocol under heat stress (35°C) was reported following 5 days of heat acclimation performing high-intensity interval work (Brade et al., 2013). Conversely, short-term repeated-sprint training under heat stress (30°C) did not influence repeated-sprint alibility in cool conditions (24°C; Petersen et al., 2010). In the current study, repeated-sprint training in both groups increased total distance in a Yo-Yo IR1 test performed in cool conditions, with a larger but non-significant effect noted in the HOT (315 m) compared with the COOL (207 m) group (Figure 2). Our data further indicate improved repeated-sprint ability in the heat in both groups, with higher mean and peak power outputs across the RST after the training interventions (Figure 5). A lower percent decrement score was also observed for mean and peak power output following the intervention in both the HOT and COOL groups. Taken together, the training intervention in both HOT and COOL conditions enhanced aerobic performance in cool conditions and repeated-sprint ability in the heat. Given the nature of the intervention (i.e., maximal sprinting), it appears that the novelty of maximal sprinting induces a similar performance enhancement, irrespective of the environmental conditions in which it is undertaken.

Interestingly, the improvement in repeated-sprint ability noted in the current study may have been dampened, as evidenced by a decrease in force production capacity (i.e., peak twitch force; Table 1) during the neuromuscular assessment Pre-RST following the intervention. This observation is suggestive of accumulated skeletal muscle fatigue due to a lack of adequate recovery between maximal exercise sessions, as a previous study demonstrated that passive heating for 11 consecutive days improved peak twitch force and MVC force production capacity in both normothermic and hyperthermic states (Racinais et al., 2017). In contrast, others have reported a failure to improve performance following short-term high-intensity training in the heat and ascribed the lack of improvement to the intense nature of such regimens. Reeve et al. (2019) demonstrated that 30 min of high-intensity interval training in the heat for 5 consecutive days reduced physiological and perceptual strain, but impaired endurance capacity during a heat stress test. It was suggested that such a regimen may not be ideal for athletes preparing to compete in the heat, as it may lead to a state of overreaching. A similar conclusion was drawn in a study where five consecutive high-intensity sessions in 30°C and 50% RH appeared to induce a state of functional overreaching (Schmit et al., 2018). These studies, along with the current data, indicate that careful consideration and monitoring of athletes must be exercised when implementing a repeated-sprint heat acclimation regimen. This may be particularly relevant in team and racquet sports, as the performance benefits stemming from repeated high and maximal intensity bouts of exercise may, in the short term, be attenuated by the accumulation of fatigue.

### Summary

A short-term repeated-sprint training intervention undertaken in HOT and COOL conditions improved Yo-Yo IR1 distance covered in cool conditions by ~23 and 16%, respectively. Repeated-sprint ability in the heat was also improved in both groups, despite the presence of residual skeletal muscle fatigue, manifested as a reduction in peak twitch force prior to the RST conducted after the training intervention. Physiological adaptations commensurate with training (e.g., lower heart rate) were evident after five sessions of repeated sprinting in the HOT and COOL groups, with partial heat adaptations (i.e., increased sweat rate, lowered *T*_sk_, and thermal sensation) emerging after training in the heat. These heat adaptations were negligible, however, as short-term repeated-sprint training improved repeated-sprint cycling ability in the heat, as well as high-intensity intermittent running performance in cool conditions, regardless of whether training was undertaken in HOT or COOL conditions.

### Perspective

Preparing to compete in the heat might be difficult for high-level team-sport athletes due to travel constraints and the time required to heat acclimate. Given that traditional heat acclimation approaches are endurance-based and require daily exposures of 60–90 min for 1–2 weeks, this study investigated whether a short-term repeated-sprint training intervention in 40°C induced adaptations commensurate with heat acclimation and greater improvements in repeated-sprint ability and aerobic performance, than training in 20°C. It was observed that five sessions of repeated-sprint training in hot and cool conditions over 7 days improved repeated-sprint ability in the heat and enhanced 20-m shuttle running performance in cool conditions in team-sport athletes. Although training in the heat initiated the emergence of some heat adaptations, the novelty and intensive nature of the brief repeated-sprint training protocol induced similar performance and physiological improvements in the hot and cool conditions. The brevity of the protocol, however, also limited recovery time and resulted in skeletal muscle fatigue. As such, fatigue should be monitored during repeated-sprint training interventions to optimize recovery and performance benefits, with an extended protocol and additional recovery between sessions, potentially allowing for greater induction of heat acclimation in similarly trained individuals. The timeframe of implementation (e.g., pre-season training or pre-competition taper) for such an intervention should also be carefully considered.

## Data Availability Statement

The raw data supporting the conclusions of this article will be made available by the authors, without undue reservation.

## Ethics Statement

The studies involving human participants were reviewed and approved by University of Canberra Human Research Ethics Committee. The patients/participants provided their written informed consent to participate in this study.

## Author Contributions

JP, OG, DP, and DB designed the study. JP and AW collected and analyzed the data. JP wrote the initial manuscript and all authors revised it and provided intellectual content. All authors contributed to the article and approved the submitted version.

### Conflict of Interest

The authors declare that the research was conducted in the absence of any commercial or financial relationships that could be construed as a potential conflict of interest.
